# Social Communication Coaching Smartglasses: Well Tolerated in a Diverse Sample of Children and Adults With Autism

**DOI:** 10.2196/mhealth.8534

**Published:** 2017-09-21

**Authors:** Neha U Keshav, Joseph P Salisbury, Arshya Vahabzadeh, Ned T Sahin

**Affiliations:** ^1^ Brain Power Cambridge, MA United States; ^2^ Psychiatry Academy Massachusetts General Hospital Boston, MA United States; ^3^ Department of Psychology Harvard University Cambridge, MA United States

**Keywords:** autism, tech, digital health, smartglasses, augmented reality, autism spectrum disorder, technology, medtech, education

## Abstract

**Background:**

Augmented reality (AR) smartglasses are an emerging technology that is under investigation as a social communication aid for children and adults with autism spectrum disorder (ASD) and as a research tool to aid with digital phenotyping. Tolerability of this wearable technology in people with ASD is an important area for research, especially as these individuals may experience sensory, cognitive, and attentional challenges.

**Objective:**

The aim of this study was to assess the tolerability and usability of a novel smartglasses system that has been designed as a social communication aid for children and adults with autism (the Brain Power Autism System [BPAS]). BPAS runs on Google Glass Explorer Edition and other smartglasses, uses both AR and affective artificial intelligence, and helps users learn key social and emotional skills.

**Methods:**

A total of 21 children and adults with ASD across a spectrum of severity used BPAS for a coaching session. The user’s tolerability to the smartglasses, user being able to wear the smartglasses for 1 minute (initial tolerability threshold), and user being able to wear the smartglasses for the entire duration of the coaching session (whole session tolerability threshold) were determined through caregiver report.

**Results:**

Of 21 users, 19 (91%) demonstrated tolerability on all 3 measures. Caregivers reported 21 out of 21 users (100%) as tolerating the experience, while study staff found only 19 out of 21 users managed to demonstrate initial tolerability (91%). Of the 19 users who demonstrated initial tolerability, all 19 (100%) were able to use the smartglasses for the entire session (whole session tolerability threshold). Caregivers reported that 19 out of 21 users (91%) successfully used BPAS, and users surpassed caregiver expectations in 15 of 21 cases (71%). Users who could communicate reported BPAS as being comfortable (94%).

**Conclusions:**

This preliminary report suggests that BPAS is well tolerated and usable to a diverse age- and severity-range of people with ASD. This is encouraging as these devices are being developed as assistive technologies for people with ASD. Further research should focus on improving smartglasses design and exploring their efficacy in helping with social communication in children and adults with ASD.

## Introduction

Modern smartglasses are small head-mounted displays that integrate a range of sensors that can capture video, audio, and movement data. Smartglasses can deliver an augmented reality (AR) experience, where the user can see virtual objects overlaid on top of their real-world view as they look through the optical display. Smartglasses delivering AR are believed to have considerable potential as educational and health care tools, and an increasingly wide range of smartglasses are available for developers and consumers [1,2].

A wide range of assistive technologies have been developed for autism spectrum disorder (ASD) including smartphone and tablet apps, computer programs, social robots, and virtual reality [3]. There have been encouraging findings about the positive impact of such technologies, yet many children and adults with ASD continue to have considerable unmet educational and health care needs. Interest has been growing in the use of AR as a teaching tool for children and adults with ASD, and understanding how people with ASD experience and are affected by head-mounted displays remain key questions that face the field [4]. An AR experience can be delivered on a variety of different platforms including smartphones, tablets, stationary displays, and on “heads-up” smartglasses. Much of the current AR research has been on AR delivered through handheld/“heads-down” devices [5-7]. Studies have demonstrated that AR delivered on smartphones, tablets, and desktop computers may help people with ASD with their attention [6], emotion recognition [8], ability to notice social cues [5], social skills [9], ability to engage in pretend play [10], and even as a navigation aid for planning trips [7]. However, using AR is not a risk-free endeavor; children using smartphone-based AR have developed postural and grip strain in addition to experiencing falls [11], and smartphone-based AR games can lead to injury through distraction, with resultant major trauma already being reported [12].

Smartglasses may offer several advantages when compared to smartphone and tablet devices and have been described as the platform of the future for AR [13]. Use of smartglasses may be less distracting and may require less cognitive workload than smartphones [14,15]. By looking through smartglasses, users can continue to look heads-up at the environment around them and also remain hands-free because smartglasses are head-worn [16]. These advantages may enable users to continue to observe the social world around them, something that is considerably impacted when using a smartphone [17]. Additionally, smartglasses allow users to keep their hands unoccupied, making it easier to use them in nonverbal communication and/or academic and occupational activities, which are particularly pertinent considerations for children and adults with ASD who demonstrate impairment in social communication [18]. To our knowledge, we have published the first report of the feasibility of using AR smartglasses to provide social and cognitive coaching in children with ASD [16].

Research is required to determine the tolerability of AR smartglasses given that ASD is accompanied by a range of sensory, behavioral, and cognitive challenges that may make wearing such devices difficult. Many people with ASD have sensory sensitivities, and they may struggle to wear conventional prescription glasses [19], brush their teeth, or comb their hair [20,21]. Smartglasses often have a similar form factor to prescription glasses, and in the case of Glass Explorer Edition (formerly known as Google Glass), may weigh the same as typical pair of prescription lenses and frame [22]. Unlike prescription glasses, smartglasses produce additional sensory stimuli in the form of visual input via their optical displays and audio via their speakers. It is therefore important to study how people with ASD respond to and tolerate wearing such devices. With the exception of conventional prescription glasses, there are only rare occasions when one would need to “wear” a face-mounted object. In this regard, wearing smartglasses may be a particularly novel experience, with few daily life comparators. This is an important consideration because people with ASD can exhibit considerable distress when exposed to unfamiliar situations, changes in routine, or changes in environment [18]. Despite the abovementioned concerns, there continues to be a dearth of research into AR smartglasses for people with ASD. The authors have found that many clinicians, educators, and people from the ASD community have expressed doubt as to whether children and adults with ASD would tolerate wearing AR smartglasses. This has led to the commonly encountered question: *but will they even wear it?* This is not surprising given that wearing conventional glasses has been highlighted as a major challenge by prominent ASD charities [23].

The importance of understanding how people with ASD will respond to such devices is heightened by the potential benefits of conducting research with smartglasses. Smartglasses, like smartphones, contain myriad sensors, such as an accelerometer and camera, and are able collect video, audio, movement, physiologic, and user interaction data [24]. These quantitative data can be collected and analyzed to undertake digital phenotyping, and more importantly, to help support research efforts to help subtype highly clinically heterogeneous behavioral conditions such as ASD [25].

To explore the tolerability of AR smartglasses, we studied whether children and adults with ASD were able to tolerate wearing the Brain Power Autism System (BPAS), novel social communication coaching smartglasses that use AR and emotional artificial intelligence [16]. BPAS has undergone feasibility [16], acceptability [26], safety [27], and clinical impact studies [28]. BPAS is based on a highly modified version of Google Glass Explorer Edition and Glass Enterprise Edition (both overseen by X Development LLC, formerly known as Google X).

## Methods

The methods and procedures of this study were approved by Asentral, Inc, Institutional Review Board, an affiliate of the Commonwealth of Massachusetts Department of Public Health.

### User Recruitment

A sequential sample of 21 children and adults with clinically diagnosed ASD were recruited from a database of individuals who completed a Web-based signup form expressing interest in participating in smartglasses research. Individuals represented a demographically and clinically diverse group comprising different ages, genders, verbal abilities, and level of functioning (Table 1). Written consent was obtained from the legal guardians of children and from cognitively able adults. Children aged 7 to 17 years provided written assent when possible. In this report, every user was accompanied by a parent or other caregiver during the session, and users and caregivers could ask for the session to stop at any time and for any reason. All users completed the Social Communication Questionnaire (SCQ) so we could document their level of social communication impairment [29]. The SCQ score demonstrates that the user sample represented a wide range of social communication impairment.

### Data Collection Procedure

**Figure 1:**
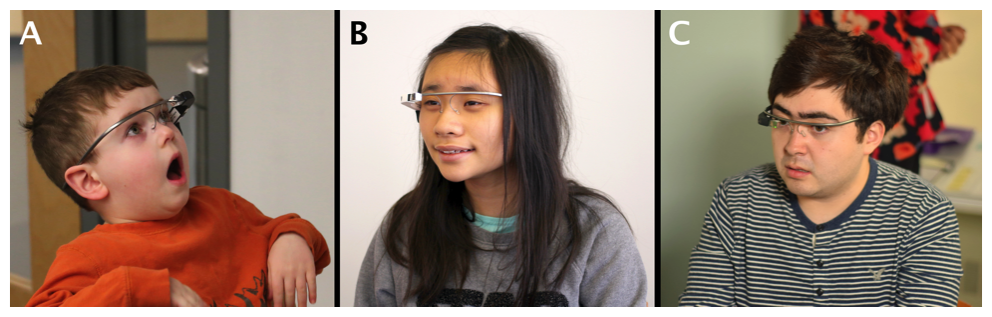
Three users with autism wearing the Brain Power Autism System and using its socioemotional coaching apps. Pictures used with user/caregiver permission.

**Figure 2:**
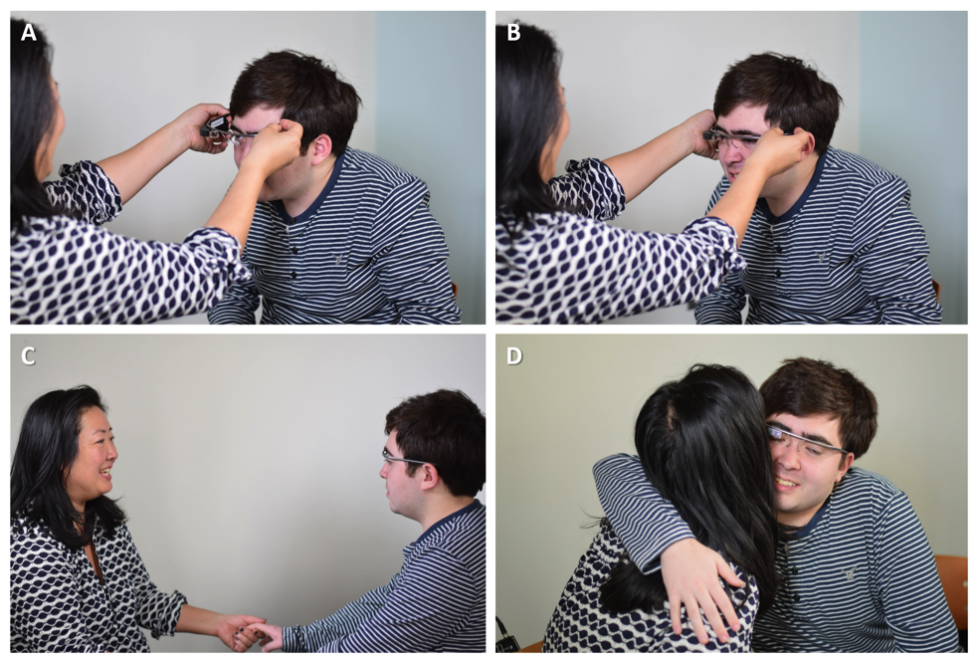
Caregiver assisting user in wearing Brain Power Autism System smartglasses during testing (A, B). User demonstrated tolerability on all 3 measures and was witnessed to spontaneously hug his caregiver during use of social communication app use (C, D). Pictures used with user/caregiver permission.

Users and caregivers were given an introductory explanation and demonstration of BPAS smartglasses. Users were then given the chance to wear the smartglasses (Figure 1), aided as needed by study staff and their caregivers for correct initial placement (Figure 2).

The user’s tolerability to the smartglasses was determined through caregiver report, the user’s ability to wear the smartglasses for 1 minute (initial tolerability threshold), and the user’s ability to wear the smartglasses for the entire duration of the coaching session (whole session tolerability threshold). The initial tolerability threshold provides a rapid understanding of how well a user would respond to the physical form factor of the smartglasses, an important consideration given the unique set of sensory and cognitive challenges of each user. The whole session tolerability threshold represents how well the user tolerates wearing the smartglasses but also represents their use of the coaching apps as they undertake a series of structured activities with their caregiver in a session lasting between 1 and 1.5 hours. At the end of the session, caregivers could rate how well they felt the user tolerated using BPAS through a 5-point Likert scale (1=very low, 5=very high). A tolerability rating of low or very low was deemed to be a negative indication of tolerability, while neutral, high, or very high caregiver ratings were noted as an indication of tolerability.

Caregivers were also asked to use a 5-point Likert scale to rate if they felt the user was able to successfully use BPAS with their assistance (1=strongly disagree, 5=strongly agree) and whether they felt the user responded more positively to BPAS smartglasses than the they had expected (1=strongly disagree, 5=strongly agree). For these responses, a higher standard had to be set compared to tolerability: a rating of agree/strongly agree (4 or 5) was determined to be a positive response for each of these questions. Users who could communicate verbally with their caregiver or study staff were asked to rate how comfortable the smartglasses were. Both caregivers and users were able to provide additional feedback to any question in the interviews.

**Table 1:** Demographics of users (N=21).

Characteristic		Mean (SD) range or n (%)^a^
Age, years, mean (SD) range		11.9 (4.9) 4.4-21.5
**Gender, n (%)**		
	Male	19 (91)
	Female	2 (10)
**Verbal, n (%)**		
	Yes	19 (91)
	No	2 (10)
Social Communication Questionnaire score, mean (SD) range		18.5 (6.1) 6-28

^a^Percentages can equal more than 100 due to rounding.

## Results

**Figure 3:**
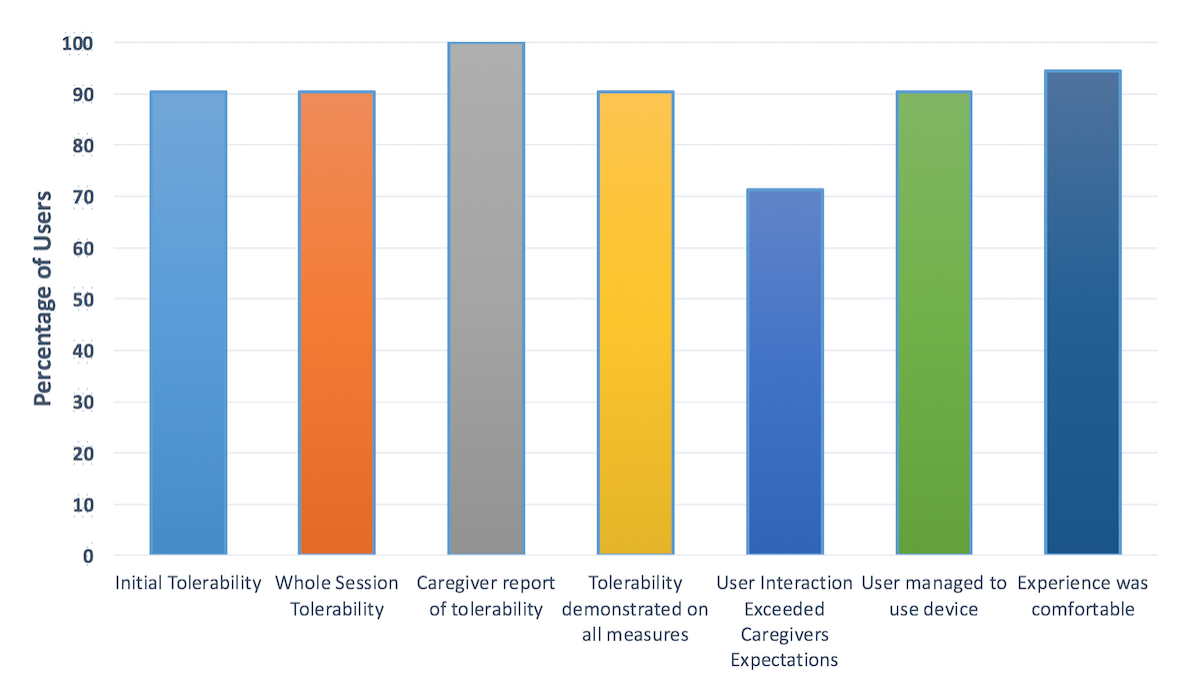
Summary of Brain Power Autism System tolerability and experience.

A total of 19 out of 21 users (91%) demonstrated tolerability on all 3 measures (caregiver report, initial tolerability threshold, and whole session tolerability threshold; Table 2). Of the 19 users who managed to pass the initial tolerability threshold (19/21, 91%), all went on to use BPAS for the entire coaching session, passing the whole session tolerability threshold (19/19, 100%). Two users, both nonverbal, did not pass the initial 1-minute tolerability threshold as they would not continue to wear the smartglasses once placed. These users were both nonverbal and were aged 5.5 and 5.75 years with SCQ scores of 25 and 28. Users who were verbal and able to answer questions (18/21) rated the smartglasses as being comfortable to use (17/18, 94%; Table 3). A majority of caregivers felt users responded more positively to the smartglasses than they had expected (15/21, 71%). A number of caregivers provided additional feedback, suggesting that users may benefit from extended and/or repeated orientation and introduction sessions with BPAS. The results are graphically represented in Figure 3.

**Table 2:** Tolerability report of Brain Power Autism System (N=21).

Tolerability measures		n (%)
**Initial tolerability threshold (worn for at least 1 minute of continuous use)**		
	Yes	19 (91)
	No	2 (10)
**Whole session tolerability threshold (worn until session completion)**		
	Yes	19 (91)
	No	2 (10)
**Caregiver report of tolerability**		
	Yes	21 (100)
	No	0 (0)
**Users demonstrating tolerability on all measures**		
	Yes	19 (91)
	No	2 (10)

**Table 3:** User experience report (N=21).

User experience responses		n (%)
**User reported that experience was comfortable (only users who were able to answer questions: n=18)**		
	Yes	17 (94)
	Mixed response/sometimes	1 (6)
	No	0 (0)
**Caregiver reported that user managed to use the device with assistance**		
	Yes	19 (91)
	Mixed response/sometimes	0 (0)
	No	2 (10)
**Caregiver reported user responded more positively to the device than expected**		
	Yes	15 (71)
	Mixed response/sometimes	6 (29)
	No	0 (0)

## Discussion

Children and adults with ASD have considerable unmet educational and behavioral health needs, and technology-aided solutions may provide a scalable and effective tool to help address these demands for resources. While AR smartglasses have been designed as a social communication aid for people with ASD [16], there is only limited research to understand how acceptable and tolerable this technology is to these individuals [16]. Our preliminary study shows that a diverse range of children and adults with ASD can tolerate wearing and using BPAS, AR smartglasses designed to function as a social communication aid for people with ASD.

Tolerability was demonstrated across all 3 of our measures in 91% of users. Every user who demonstrated initial 1-minute tolerability managed to continue to demonstrate tolerability for the entire coaching session that ran between 1 and 1.5 hours. This suggests that the tolerability of users with ASD to such smartglasses can be accurately predicted based on their ability to initially use the device over a relatively short amount of time. The 2 users who did not tolerate wearing the device were both nonverbal, younger in age, and had greater social impairment as highlighted by their higher SCQ scores. Further investigation is warranted to determine how to improve tolerability in younger children with ASD who have greater language and social communication challenges.

Our data help to answer our initial question: *but will they even wear it?* In our experience, this is one of the most common questions that parents, educators, and researchers ask us when BPAS is shown to them. These data show that children and adults with ASD can not only wear smartglasses for relatively lengthy durations of time but are able to use them and describe the experience as comfortable. How the users interacted with BPAS surpassed the expectations of their caregivers in most cases. We did find that both nonverbal users struggled to wear the smartglasses and were unable to pass the initial tolerability threshold. Based on feedback from caregivers, a more gradual introduction and orientation process to the smartglasses may have been useful in these cases.

Additionally, given that sensor-rich smartglasses are quantitative data gathering tools, it is helpful to know that they can be worn for such durations in people with behaviorally heterogeneous conditions that could benefit from digital phenotyping and subtyping.

While our results are promising, there are a number of limitations. Although this work is, to our knowledge, the first report of the tolerability of smartglasses as a social communication aid in people with ASD, our sample size is moderate (N=21). Additionally, given the customized nature of BPAS, generalizability may be limited in the case of other smartglasses, different smartglasses software apps, and even an unmodified Google Glass device.

More longitudinal research would be useful to determine whether the tolerability that we have observed continues to last after repeated coaching sessions. Understanding the effects of repeated sessions over a longer duration of time is important, as many training and educational programs for people with ASD involve repeated sessions over a long period of time. Further research is required to investigate the efficacy of AR smartglasses in ASD, but the tolerability and usability of such devices does not appear to be a substantial barrier to their use.

## Abbreviations

AR: augmented reality
ASD: autism spectrum disorder
BPAS: Brain Power Autism System
SCQ: Social Communication Questionnaire
